# Public responses and parasocial relationships following senator John Fetterman’s depression disclosure

**DOI:** 10.3389/fpubh.2024.1393257

**Published:** 2024-10-08

**Authors:** Jessica Gall Myrick, Jessica Fitts Willoughby

**Affiliations:** ^1^Department of Media Studies, The Pennsylvania State University (PSU), University Park, PA, United States; ^2^Department of Strategic Communication, Washington State University, Pullman, WA, United States

**Keywords:** depression, emotion, media, politics, attention, information seeking

## Abstract

Research has found that when a public figure discloses an illness, it can motivate members of the public to reconsider their own health behaviors, particularly when they have a parasocial relationship with the public figure. When the public figure is a politician, it is possible that partisan differences may also influence emotional, attentional, and behavioral responses to health news. We empirically examined public responses to Democrat John Fetterman’s disclosure of his treatment for depression shortly after he was inducted into the United States Senate as the junior senator from Pennsylvania in 2023. Using a survey of adult Pennsylvania residents who identified as eligible voters in April 2023, we found that 204 respondents were aware of the news of Fetterman’s diagnosis of and treatment for depression. Our data revealed that differences in demographics and parasocial relationships—both positive and negative—with Fetterman predicted different patterns of emotional responses to the news. In addition, age, anger, a negative parasocial relationship, and a positive parasocial relationship were associated with additional outcomes, including attention to news about the disclosure and depression-related information seeking. Mental health advocates could use politicians’ depression disclosures to provide information at a time when people are paying more attention to the condition; however, they may need to find other public figures to counteract negative responses to partisan officials. Policymakers could also consider funding mental health campaigns, which could be launched alongside public figure disclosures.

## Introduction

The psychological attachments people form with public figures through media can influence their responses to learning about a public figure’s illness (1). These psychological attachments manifest in various ways, but often they appear as parasocial relationships, where individuals experience a one-sided sense of familiarity and emotional attachment to a public figure represented in the media (2). For instance, learning about singer Demi Lovato’s diagnosis of bipolar disorder resulted in a decrease in negative stereotypes and social distancing intentions amongst audiences. This change in attitude was partially due to the influence of an existing parasocial relationship with Lovato (3).

In general, health disclosures by celebrities and public figures can impact emotions, information-seeking behaviors, and discussions about health on social media and can cause changes in health behavior, increased news coverage, and, in some cases, policy changes (4). Indeed, in a meta-analysis of 14 studies with more than 5,700 participants that focused on the impact of health disclosures by celebrities on audience involvement and behavioral intentions, Kresovich and Noar (5) found that there was a small- to medium-sized positive association between audience involvement and behavioral intentions related to celebrity health disclosures. However, many of these studies have examined disclosures by generally popular entertainers. The present study aimed to assess whether similar public responses occur when a partisan political figure discloses a stigmatized health condition.

Through repeated media exposure to public figures, individuals can respond like a friend (i.e., develop a positive parasocial relationship) or a well-known enemy (i.e., develop a negative parasocial relationship) when misfortune befalls the public figures and is reported in the news (6). Compared to widely popular celebrities, politicians are often strongly liked or disliked by partisans who identify with different political groups. As such, their illness disclosures could have different effects on and could evoke different responses from the public. For instance, Myrick and Chen (7) found that many Americans experienced schadenfreude, or pleasure in another’s pain, when they learned about conservative media figure Rush Limbaugh’s diagnosis of lung cancer in February 2020 and about Kentucky Republican Senator Rand Paul’s COVID-19 diagnosis in March of 2020.

In both cases, parasocial relationships—both positive and negative—with these political figures increased feelings of schadenfreude, which in turn decreased audience intentions to take preventative health measures (e.g., avoiding smoking in response to Limbaugh’s lung cancer or minimizing social contact during the COVID-19 pandemic in response to Paul’s COVID-19). Interestingly, feelings of schadenfreude and anxiety after learning about Limbaugh’s lung cancer prompted participants to seek more information about the disease; however, these same emotions in response to Paul’s COVID-19 diagnosis did not encourage individuals to seek more information about the virus.

These results reveal that although political figures disclosing an illness can shape public emotions and information-seeking behaviors, these responses are not uniform and vary depending on the health condition or the politician involved. We know less about how the public responds to the news of a politician’s mental health crisis, especially given the stigma surrounding mental health (8). Understanding the effects of such a disclosure is an important public health goal because news media are often identified as both sources of mental health information and perpetrators of stigmatizing stereotypes that prevent some people from seeking treatment (9, 10).

If a politician reveals a mental illness diagnosis, it could make their constituents feel compassion or other tender emotions, which scholars define as emotions related to meaningful or moral events and caring for others (11). Researchers have found that news stories about members of stigmatized groups can elicit tender emotions (e.g., compassion, sympathy), which in turn encourages intentions to help and seek more information (12, 13). Public figure health disclosures have long been associated with information seeking. For instance, after President Ronald Reagan had part of his colon removed, calls to cancer information lines about colon cancer increased significantly (14). However, if Fetterman’s mental health disclosure had caused individuals who already disliked him to experience anger or schadenfreude, then negative outcomes must also be considered. Negative emotions, such as anger, experienced after receiving the news can prompt defensive audience responses (15), and anger has also been linked with lower levels of politics-related information seeking (16).

To investigate these possibilities, we empirically examined public responses to Democrat John Fetterman’s disclosure of his treatment for depression shortly after he was inducted into the United States Senate as the junior senator from Pennsylvania in 2023. This disclosure came after he had suffered a stroke during his election campaign, after which he spoke publicly about the stroke and its effects, stating that he felt he was recovering well and would be well-poised to serve (17). The difficulty he experienced in processing information following the stroke was cited as a potential cause of the depression (18).

To summarize, research has shown that public figure health disclosures can affect audience responses; however, the responses may differ based on the strength and type of the parasocial relationship and emotional responses to the disclosure news. Moreover, when a disclosed health condition is stigmatized and associated with a partisan public figure, there could be further differences in public responses compared to disclosures involving other people or other health conditions. The present study can help determine if sociodemographic similarities (e.g., race, gender, and political party) are more or less predictive than parasocial relationships. Based on previous literature, we formulated two hypotheses and asked two research questions:

*H1:* Respondents who share some similarities with Fetterman (gender, race, political party, having a depression diagnosis, or knowing someone with a depression diagnosis) will report stronger tender emotions (e.g., concern or compassion) and lower negative emotions (e.g., schadenfreude or anger) to the news about Fetterman’s depression diagnosis.

*H2:* After controlling for demographic variables, respondents’ parasocial relationships—both negative and positive—will predict stronger emotional responses (concern, compassion, schadenfreude, or anger) to the news about Fetterman’s depression diagnosis.

*RQ1:* Which variables—demographics, parasocial relationships, or emotional responses—will be the strongest predictors of post-disclosure attention to news about Fetterman’s diagnosis and treatment?

*RQ2:* Which variables—demographics, parasocial relationships, or emotional responses—will be the strongest predictors of post-disclosure depression information seeking?

## Methods

### Procedure

We used Cloud Research’s Prime Panels to recruit respondents for this online survey from 6 April 2023 to 10 April 2023. The eligibility criteria included Pennsylvania residents who were at least 18 years of age, U.S. citizens, and eligible to vote. We selected this population because they were Fetterman’s constituents and potentially more likely to consume news about him, given their voter eligibility status. After excluding individuals who failed attention checks, who did not report their age, or who did not report their citizenship status, 352 respondents were included in this study.

The respondents were first shown an image of Fetterman (his official U.S. Senate photograph) and asked to respond to questions assessing their parasocial relationship with him. Then, the respondents read the following statement: “John Fetterman was sworn in as Pennsylvania’s junior senator in January of 2023. About a month later, in the middle of February, John Fetterman voluntarily checked himself into Walter Reed Hospital, at the advice of the Senate physician, in order to receive treatment for severe depression.” The respondents were then asked: “Prior to taking part in this survey, were you aware that John Fetterman had entered the hospital this year to be treated for severe depression?” Nearly three-fifths (58.0%) said “yes,” while 37.2% said “no,” and another 4.8% chose “not sure/cannot remember.” Those who said yes (*N* = 204) were the focus of the subsequent analyses.

### Participants

Of the 204 respondents who had heard of Fetterman’s depression diagnosis, 33.8% identified as Republicans, 41.7% as Democrats, 18.6% as Independents, and 5.9% as members of other parties. The majority of the respondents (60.3%) identified as women, 38.7% identified as men, and 1.0% identified as non-binary. The average age of the respondents was 49.16 years (*SD* = 17.28, range: 18–80 years). Nearly two-fifths (37.7%) of the respondents reported having been diagnosed with depression at some point in their lives, while half (51.5%) of the respondents reported having a close friend or family member with depression.

### Measures

All items were measured using 7-point Likert-type scales, unless otherwise noted. See Table 1 for correlations between the study variables.

**Table 1:** Bivariate correlation matrix.

	1	2	3	4	5	6	7	8	9	10	11	12
1. Democrat												
2. White	−0.21**											
3. Man	−0.08	−0.03										
4. Age	−0.14	0.28**	0.07									
5. Has depression	0.18**	0.12	−0.12	−0.23**								
6. Someone close has depression	0.14*	0.10	−0.15*	−0.20**	0.45**							
7. Positive PSR	0.56**	−0.15*	−0.01	−0.19**	0.18**	0.19**						
8. Negative PSR	−0.45**	0.02	0.06	−0.00	−0.10	−0.16*	−0.59**					
9. Post-disclosure anger	−0.15*	−0.04	0.17*	−0.11	−0.06	−0.10	0.00	0.39**				
10. Post-disclosure schadenfreude	0.02	−0.12	0.09	−0.23**	0.01	−0.03	0.13	0.36**	0.48**			
11. Post-disclosure tender emotions	0.44**	−0.06	−0.08	−0.11	0.20**	0.24**	0.76**	−0.57**	0.04	0.02		
12. Attention	0.34**	−0.07	0.94	−0.06	0.16*	0.19**	0.54**	−0.30**	0.12	0.07	0.50**	
13. Seeking information	0.14*	−0.11	−0.00	−0.26**	0.10	0.03	0.22**	0.06	0.25**	0.23**	0.15*	0.19**

*p < 0.05, **p < 0.01 (2-tailed). Democrat: 0 = not a Democrat and 1 = Democrat; White: 0 = not White and 1 = White; Man (gender): 0 = not a man and 1 = a man; Depression diagnosis: 0 = no diagnosis and 1 = has been diagnosed; and Knows someone with depression: 0 = does not know anyone with depression and 1 = knows at least one person. Pos. PSR = positive parasocial relationship and Neg. PSR = negative parasocial relationship.

#### Parasocial relationships

A total of 22 items comprising the positive parasocial relationship (PSR) scale (11 items) and the negative parasocial relationship scale (11 items), answered on scales from 1 = strongly disagree to 7 = strongly agree, were adopted from Hartmann et al. (6). An exploratory factor analysis (EFA) with Promax rotation revealed two separate factors: one for the 11-item negative PSR scale (Cronbach’s *α* = 0.96, *M* = 3.51, *SD* = 1.93), explaining 58.77% of the variance, and one for the 11-items positive PSR scale (Cronbach’s α = 0.96; *M* = 3.69, *SD* = 1.82), explaining 16.59% of the variance.

#### Post-disclosure emotions

The respondents were presented with the following prompt: “Please think about when you first learned of John Fetterman’s severe depression and admittance to the hospital. How did you feel? ‘When I found out John Fetterman was diagnosed with depression and entered the hospital for treatment, I felt _________.’” This was followed by 14 emotions, answered on a scale from 1 = not at all to 7 = very much. The EFA with Promax rotation revealed that the word upset was cross-loaded onto multiple factors. The item upset was removed, and the subsequent EFA revealed three factors. The largest factor, explaining 40.64% of the variance, included the tender emotions, such as “sympathetic,” “compassionate,” “concerned,” “hopeful,” “worried,” “sad,” “optimistic,” “stunned,” and “surprised” (Cronbach’s *α* = 0.91; M = 4.34 and SD = 1.56). The second factor, explaining 21.10% of the variance, comprised the two anger words (“aggravated” and “angry,” *r* = 0.72, *p* < 0.001; M = 2.76 and SD = 1.88). The third factor, explaining an additional 9.18% of the variance, included “pleased” and “secretly happy,” which we labeled as schadenfreude (*r* = 0.70, *p* < 0.001; M = 2.36 and SD = 1.70).

#### Attention to Fetterman’s depression news

The respondents were asked: “After you first learned about John Fetterman’s severe depression, how much attention did you pay to stories in the media (in any form, print, television, or online) or on social media about it?” The endpoints and midpoint of the 7-point Likert-type scale were labeled as 1: “None after I initially heard about it,” 4: “Not a little, but not a lot of attention,” and 7: “A great deal, I read or watched everything I could on the topic” (M = 4.34 and SD = 1.57).

#### Depression-related information seeking

The respondents were asked to report if they sought any information from any of the following sources after initially learning about Fetterman’s depression diagnosis and hospital admittance: an online search engine (e.g., Google, Bing) (24.5%); social media (e.g., TikTok, Instagram) (18.1%); a healthcare provider (e.g., a nurse or a doctor) (12.3%); a friend or family member (18.1%); a specific medical website (e.g., Mayo Clinic, CDC, etc.) (14.7%); and a specific news source (e.g., CNN, Fox, MSNBC, New York Times, etc.) (24%). A summative index was formed if the box was checked and the value was coded as 1, with higher numbers indicating that more sources were used to seek information about depression (M = 1.12, SD = 1.19, and mode = 1). The index was kurtotic (K = 5.42); 59 out of the 204 respondents did not seek depression information. Hence, we converted the index into a binary index with 0 “for not searching” and 1 “for searching through any medium.”

## Results

H1 and H2 predicted that demographics, including political party affiliations, would be related to emotional responses to Fetterman’s depression disclosure. Three linear regressions with hierarchical entry (block entry) were run, with the demographic variables, such as gender, race, political party, and age, in the first block, a personal depression diagnosis and knowing someone with depression in the second block, and a positive PSR and a negative PSR in the third block, with tender emotions, schadenfreude, and anger as separate outcome variables (see Table 2).

**Table 2:** Linear regression with hierarchical entry predicting emotional responses to depression disclosure.

	Schadenfreude			Anger			Tender emotions		
	Model 1	Model 2	Model 3	Model 1	Model 2	Model 3	Model 1	Model 2	Model 3
Democrat	−0.03	−0.02	0.03	−0.15*	−0.14	−0.08	0.44***	0.41***	−0.01
White	−0.07	−0.06	−0.01	−0.03	−0.02	0.02	0.05	0.01	0.02
Man	.0.12	0.11	0.08	0.17*	0.16*	0.13*	−0.04	−0.01	−0.05
Age	−0.23**	−0.25**	−0.16*	−0.12	−0.14	−0.07	−0.06	−0.01	0.03
With a depression diagnosis		−0.01	−0.04		0.00	−0.03		0.06	0.03
Knows someone with depression		−0.06	−0.03		−0.08	0.506		0.15*	0.07
Pos. PSR			0.47***			0.37***			0.65***
Neg. PSR			0.64***			0.55***			−0.18**
*R^2^*	0.077	0.081	0.348	0.063	0.069	0.259	0.203	0.232	0.612
*R^2^* change			0.267***		0.005	0.191***		0.028	0.380***

*p < 0.005; **p < 0.01; and ***p < 0.001. Coefficients are standardized. Democrat: 0 = not a Democrat and 1 = Democrat; White: 0 = not White and 1 = White; Man (gender): 0 = not a man and 1 = a man; Depression diagnosis: 0 = no diagnosis and 1 = has been diagnosed; and Knows someone with depression: 0 = does not know anyone with depression and 1 = knows at least one person. Pos. PSR = positive parasocial relationship and Neg. PSR = negative parasocial relationship. Values in bold are significant at *p* < 0.05.

The final model with all three blocks predicting schadenfreude was significant, with *F* (8, 193) = 12.86, *p* < 0.001, and adjusted *R*^2^ = 0.35. Age (*β* = −0.16, *b* = −0.02, 95% CI [−0.028 and − 0.003], and *p* = 0.016), a positive PSR (*β* = 0.47, *b* = 0.43, 95% CI [0.286 and 0.577], and *p* < 0.001), and a negative PSR (*β* = 0.64, *b* = 0.55, 95% CI [0.427 and 0.681], and *p* < 0.001) were the significant predictors of schadenfreude in the model. Notably, the final block added a significant amount of variance, demonstrating the importance of both positive PSR and negative PSR in predicting schadenfreude.

The final model with all three blocks predicting anger was also significant, with *F* (8, 193) = 8.45, *p* < 0.001, and adjusted *R*^2^ = 0.23. Gender (man) (*β* = 0.13, *b* = 0.49, 95% CI [0 0.013 and 0.970], and *p* = 0.044), a positive PSR (*β* = 0.37, *b* = 0.381, 95% CI [0.207 and 0.555], and *p* < 0.001), and a negative PSR (*β* = 0.55, *b* = 0.535, 95% CI [0.382 and 0.687], and *p* < 0.001) were the significant predictors of anger. Regarding schadenfreude, the third block, which included the two PSR variables, added a significant amount of variance.

The final model with all three blocks predicting the tender emotions was significant, with *F* (8, 192) = 37.80, *p* < 0.001, and adjusted *R*^2^ = 0.60. Only a positive PSR (*β* = 0.65, *b* = 0.56, 95% CI [0.454 and 0.665], and *p* < 0.001) and a negative PSR (*β* = −0.18, *b* = −0.14, 95% CI [−0.235 and − 0.051], and *p* = 0.002) were the significant predictors of the tender emotions. In addition, the third block added a significant amount of variance.

RQ1 asked which variables would predict post-disclosure attention to the news about Fetterman’s diagnosis and treatment. A linear regression model with hierarchical entry was run to predict news attention, with the demographic variables in Block 1, depression diagnosis or knowing someone with depression in Block 2, the two types of PSR in Block 3, and the three emotion groups in Block 4. The final model with all four blocks was significant, with *F* (11, 189) = 9.12, *p* < 0.001, and adjusted *R*^2^ = 0.31 (Table 3). In the final model with all four blocks, a positive PSR (*β* = 0.37, *b* = 0.31, 95% CI [0.312 and 0.492], and *p* < 0.001) was the only significant predictor of this outcome, although knowing a close friend or family member (*β* = 0.11, *b* = 0.35, 95% CI [−0.063 and 0.772], and *p* = 0.096), anger (*β* = 0.12, *b* = 0.10, 95% CI [−0.018 and 0.222], and *p* = 0.095), and the tender emotions (*β* = 0.18, *b* = 0.18, 95% CI [−0.015 and 0.364], and *p* = 0.071) had *p*-values under 0.10.

**Table 3:** Linear regression with hierarchical entry predicting attention to depression disclosure news.

	Model 1	Model 2	Model 3	Model 4
Democrat	0.34***	0.31***	0.06	0.07
White	0.01	−0.04	−0.02	−0.03
Man	0.03	0.06	0.03	0.02
Age	−0.01	0.05	0.10	0.10
Depression diagnosis		0.08	0.06	0.05
Knows someone with depression		0.16*	0.12	0.11
Pos. PSR			0.53***	0.37***
Neg. PSR			0.06	0.03
Anger				0.12
Schadenfreude				0.00
Tender emotions				0.18
*R^2^*	0.116	0.153	0.318	0.347
*R^2^* change		0.037*	0.165***	0.029*

*p < 0.05 and ***p < 0.001. Democrat: 0 = not a Democrat and 1 = Democrat; White: 0 = not White and 1 = White; Man (gender): 0 = not a man and 1 = a man; Depression diagnosis: 0 = no diagnosis, 1 = has been diagnosed; and Knows someone with depression: 0 = does not know anyone with depression and 1 = knows at least one person. Pos. PSR = positive parasocial relationship and Neg. PSR = negative parasocial relationship. Values in bold are significant at *p* < 0.05.

RQ2 asked which variables would predict post-disclosure information seeking about depression. A binomial logistic regression found that older age decreased the likelihood of seeking information about depression by 3% (odds ratio = 0.97), while a positive PSR (58%; odds ratio = 1.58), a negative PSR (45%; odds ratio = 1.45), and post-disclosure anger (34%; odds ratio = 1.34) all increased the odds of information seeking (Table 4). No other variables in the model were significant.

**Table 4:** Logistic regression predicting depression-related information seeking.

Predictor				95% CI for Exp(B)	
	B	*p*	Exp(B)	*LL*	*UL*
Democrat	0.607	0.200	1.835	0.726	4.640
White	−0.197	0.772	0.821	0.218	3.099
Man	0.066	0.862	1.068	0.952	2.234
Age	**−0.027**	**0.016**	**0.973**	**0.510**	**0.995**
Depression diagnosis	0.167	0.691	1.182	0.520	2.687
Knows someone with depression	−0.238	0.552	0.788	0.360	1.726
Pos. PSR	**0.454**	**0.014**	**1.575**	**1.094**	**2.266**
Neg. PSR	**0.372**	**0.029**	**1.451**	**1.039**	**2.026**
Anger	**0.292**	**0.017**	**1.340**	**1.053**	**1.705**
Schadenfreude	0.065	0.676	1.067	0.767	1.446
Tender emotions	0.073	0.644	1.076	0.789	1.467
Constant	−1.698	0.241	0.183	**–**	**–**

Exp(B) are the odds ratios, CI = confidence interval; *LL* = lower limit; and *UL* = upper limit. Democrat: 0 = not a Democrat and 1 = Democrat; White: 0 = not White and 1 = White; Man (gender): 0 = not a man and 1 = a man; Depression diagnosis: 0 = no diagnosis and 1 = has been diagnosed; and Knows someone with depression: 0 = does not know anyone with depression and 1 = knows at least one person. Values in bold are significant at *p* < 0.05.

## Discussion

Our data lend support to the notion that a politician can be a polarizing figure while simultaneously bring attention to and evoke compassionate feelings about people living with depression. Both positive parasocial relationships, akin to a mediated friendship, and negative parasocial relationships, akin to having an enemy or a disliked colleague, increased the antisocial emotional responses to Fetterman’s depression disclosure (schadenfreude and anger) and information-seeking behavior about depression. However, only a positive parasocial relationship was associated with increased tender emotions, such as compassion and concern. Importantly, a positive parasocial relationship with Fetterman was related to people paying more attention to additional news about his depression. Paying attention beyond the initial disclosure news is beneficial from a public health perspective as it means that audiences may learn more about treatment options and their effectiveness, thereby helping to counteract the common stigmatizing belief that mental illness is not treatable (19).

Interestingly, anger was associated with information seeking about depression; this finding was in contrast with the findings of some previous studies linking anger to decreased information seeking about politics (16). Anger, unlike other negative emotions, is an approach emotion associated with risk-taking and action (20). People who had a positive parasocial relationship with Fetterman might have felt anger when others mocked him or expressed schadenfreude (as we found some people did), while others might have felt anger because they perceived him as not doing his job or as unfit for the job. Given that Fetterman is not up for reelection until 2028 and has no announced opponent for the distant election, it could be that the angry respondents discovered that information seeking about depression was one way to channel their emotions. However, they might have been seeking information in an effort to reinforce their existing views (depression can be treated vs. depression makes one unfit). More research is needed to investigate this possibility.

In our analysis, the respondents’ political party affiliations were not a significant predictor of emotional responses, attention to subsequent news, or information seeking. This shows that partisanship may not be a simple sociodemographic factor for segmenting audiences for mental health messaging after a politician discloses a diagnosis. Contrarily, parasocial relationships (especially a positive one) were more strongly associated with our outcomes. Research across multiple media and contexts involving public figures has shown that additional, or repeated, exposure can strengthen parasocial relationships (21, 22). As such, it could be that frequent political news consumers are more likely to be moved by a politician’s depression disclosure than the partisans of the same party.

This study has certain limitations. It included a small sample collected in relation to one (somewhat eccentric) senator who also had a recent stroke that was highly publicized. We do not know how the results might change with a different sample based on a depression disclosure from a different senator from a different political party and/or with a different personality or health history. In addition, we did not assess general information-seeking tendencies or general media, which future research could consider as covariates.

Future research can build on our findings to assess how information seeking prompted by a politician’s depression disclosure relates to stigma and eventual behaviors, be it advocacy for others or finding treatment for oneself. Another avenue for future research is to use experiments in which participants are presented with hypothetical disclosures from a variety of politicians to better understand the issues of causality, which our cross-sectional data could not show. Responses to political figures’ disclosures versus celebrities’ or other public figures’ disclosures could also be examined to compare how differences in the source might impact results. In addition, future research could benefit from examining how mental health advocates and other interested organizations can capitalize on opportunities presented by media coverage of mental health disclosures by politicians, without inadvertently alienating certain subgroups of the general public.

This work also has implications for policies. Despite the growing awareness of the importance of mental health in the United States, there are a number of barriers to finding treatment (23). Policies are needed to support structural resources for those in need of mental healthcare so that when events such as public figure disclosures prompt information seeking, people are able to access trustworthy information and resources. In addition, with the plethora of information available online, some of which is inaccurate or deceitful, it is important for organizations with medically accurate information and resources to position themselves in ways that brings them to the top of search results. Future research can also employ these findings to target timing and audience segmentation.

## Conclusion

In conclusion, we found that Fetterman’s depression was associated with emotional responses and information behaviors, such as paying more attention to subsequent news and seeking out information about depression. These responses differed, slightly, for people who already felt positively or negatively connected to the Senator. Mental health advocates could use politicians’ depression disclosures to provide information at a time when already sympathetic audiences are paying more attention. However, they may need to highlight other public figures to counteract the stigma generated by partisan opponents.

## Data Availability

The raw data supporting the conclusions of this article will be made available by the authors, without undue reservation.
